# The complex interaction between anxiety and cognition: insight from spatial and verbal working memory

**DOI:** 10.3389/fnhum.2013.00093

**Published:** 2013-03-28

**Authors:** Katherine E. Vytal, Brian R. Cornwell, Allison M. Letkiewicz, Nicole E. Arkin, Christian Grillon

**Affiliations:** ^1^Section on Neurobiology of Fear and Anxiety, National Institute of Mental Health, National Institutes of HealthBethesda, MD, USA; ^2^Faculty of Life and Social Sciences, Swinburne University of TechnologyHawthorn, VIC, Australia

**Keywords:** anxiety, working memory, cognition, startle, electromyography, performance

## Abstract

Anxiety can be distracting, disruptive, and incapacitating. Despite problems with empirical replication of this phenomenon, one fruitful avenue of study has emerged from working memory (WM) experiments where a translational method of anxiety induction (risk of shock) has been shown to disrupt spatial and verbal WM performance. Performance declines when resources (e.g., spatial attention, executive function) devoted to goal-directed behaviors are consumed by anxiety. Importantly, it has been shown that anxiety-related impairments in verbal WM depend on task difficulty, suggesting that cognitive load may be an important consideration in the interaction between anxiety and cognition. Here we use both spatial and verbal WM paradigms to probe the effect of cognitive load on anxiety-induced WM impairment across task modality. Subjects performed a series of spatial and verbal *n*-back tasks of increasing difficulty (1, 2, and 3-back) while they were safe or at risk for shock. Startle reflex was used to probe anxiety. Results demonstrate that induced-anxiety differentially impacts verbal and spatial WM, such that low and medium-load verbal WM is more susceptible to anxiety-related disruption relative to high-load, and spatial WM is disrupted regardless of task difficulty. Anxiety impacts both verbal and spatial processes, as described by correlations between anxiety and performance impairment, albeit the effect on spatial WM is consistent across load. Demanding WM tasks may exert top-down control over higher-order cortical resources engaged by anxious apprehension, however high-load spatial WM may continue to experience additional competition from anxiety-related changes in spatial attention, resulting in impaired performance. By describing this disruption across task modalities, these findings inform current theories of emotion–cognition interactions and may facilitate development of clinical interventions that seek to target cognitive impairments associated with anxiety.

## Introduction

Anxiety disorders are more prevalent than any other mental health disorder, composing the majority of lifetime mental health disorders worldwide (Kessler et al., 2009). Given this, the study of anxiety is a critical public health issue because it places a considerable emotional, social, and financial burden on both the individual and society as a whole. Along with the emotional facets of the disorder, anxiety patients have difficulty concentrating and report feeling distracted, which in turn can negatively impact their job performance and interpersonal relationships. One popular hypothesis is that working memory (WM) plays a key role in the cognitive problems experienced by anxious people by limiting resources necessary to perform goal-directed tasks (Eysenck and Calvo, 1992; Eysenck, 1998; Shackman et al., 2006; Vytal et al., 2012). Despite difficulties with replicating anxiety-related impairment in the lab (Fales et al., 2008; Porcelli et al., 2008; Qin et al., 2009) WM capacity and performance is shown to be significantly reduced in patient populations (Lucas et al., 1991; Boldrini et al., 2005) and individuals with trait anxiety (Darke, 1988; Eysenck, 1998). WM is central to healthy functioning because it supports online maintenance and manipulation of information (e.g., carrying on a conversation, or tallying the cost of a grocery bill while shopping). Cognitive disruption in anxiety is thought, in part, to reflect the presence of an attentional bias (Robinson et al., under review), where anxiety takes the reins of certain sensory, perceptual, and attentional processes, and threatening information is preferentially processed over other potentially important information (for a meta-analytic review of attentional bias in anxiety see Bar-Haim et al., 2007).

Anxiety's influence on behavior encompasses changes in early perceptual processes as well as changes in higher-order cognitive processes later downstream. Anxiety alters early sensory-perceptual processes in the auditory (Cornwell et al., 2007) and visual system (Lim et al., 2009; Shackman et al., 2011) that may serve to promote threat detection (e.g., detection of auditory tones or visual cues), and this garnering of resources extends into cognitive-affective biases that are manifested in behavior. Examples of this are found in studies where negatively valenced stimuli are processed more rapidly under anxious conditions (Robinson et al., 2011, 2012). However, this bias may be detrimental to other goal-directed behaviors that are not threat-relevant. As such, performance on tasks that involve attention, maintenance of information, and rapid sensory perception may be impaired.

Further impairment may result from additional competition for resources, this time at the level of executive processes. There are several theories [e.g., processing efficiency (Eysenck and Calvo, 1992), two-component model (Vytal et al., 2012), and hemispheric asymmetry hypothesis (Shackman et al., 2006)] that have built upon this basic premise, and although they are not necessarily mutually exclusive, they make different predictions about the influence of anxiety on cognition. One important distinction that underlies each of these theories is that anxiety can be described by both anxious arousal (e.g., physiological changes in heart-rate variability and eccrine responses, increased vigilance, and priming of other sensory-dependent defensive mechanisms) and anxious apprehension (e.g., awareness of physiological changes, worry, and rumination) (Heller et al., 1997). These two components rely on separable neural systems (Nitschke et al., 1999). In a similar vein, although verbal and spatial WM share many neural resources, they also engage separable neural systems, some of which overlap with the systems above [e.g., anxious apprehension and verbal WM engage dorsal, medial, and ventral prefrontal cortex (PFC) (D'Esposito et al., 1998; Kalisch et al., 2006; Engels et al., 2007; Paulesu et al., 2010), anxious arousal and spatial WM engage unique regions in middle and ventral PFC (Clark et al., 2003; Dalton et al., 2005; Silk et al., 2010), for a meta-analysis of spatial and verbal WM neuroimaging studies see Owen et al., 2005]. As such, although both components of anxiety (anxious apprehension and anxious arousal) are likely to affect any type of WM, they may differentially disrupt verbal and spatial WM. Specifically, anxious apprehension and anxious arousal may preferentially disrupt verbal and spatial WM, respectively. This is because verbal WM processes may share more neural circuitry with anxious apprehension (e.g., mechanisms involved in verbal information encoding and verbal-based worry) and spatial WM may share more neural circuitry with anxious arousal (e.g., mechanisms involved in spatial attention).

Based exclusively on the anxious apprehension component, processing efficiency theory proposes that anxious worry reduces WM capacity in general by competing for executive resources; the greater the worry and the more difficult the task, the greater the disruption (Eysenck and Calvo, 1992). This claim is based on the proposal that worry reduces decreases processing efficiency and increases the amount of effort necessary to perform a task. Increased effort is reflected in *increased RT*, not performance impairment. Alternatively, the two-component model claims that anxious apprehension disrupts WM performance *accuracy*, and that this disruption is greatest when WM tasks are *easy* because there are free resources for anxious apprehension to engage. Further, the two-component model proposes a differential effect of anxiety on verbal versus spatial WM based on competition for a separate combination of resources. The assertion is that high-load verbal WM impairment abolishes the impact of anxiety by engaging top-down emotional control mechanisms (similar to those involved in explicit emotion regulation). In contrast, high-load spatial WM impairment persists, in part because of resource competition with the priming of defensive mechanisms (e.g., perceptual sensitivity, autonomic arousal), which unlike anxious apprehension is sustained regardless of WM load. Finally, others (Shackman et al., 2006) have proposed that anxiety uniquely disrupts spatial WM performance accuracy, because task-irrelevant anxious arousal components and spatial WM processes compete for resources in the right PFC and other more posterior regions (e.g., intraparietal sulcus, posterior parietal cortex). Support for all three theories has been found (see Eysenck and Calvo, 1992) for a review of support for processing efficiency, Vytal et al., 2012 for support of the two component model, and Lavric et al., 2003; Shackman et al., 2006 for findings in line with the hemispheric asymmetry proposal, however, no single study has ever directly compared support for all three theories by combining both task modality (i.e., spatial and verbal) and cognitive load (i.e., task difficulty). Previous research has come close (Shackman et al., 2006), but psychometric differences in low-load tasks prevented explicit evaluation of these two factors.

Recently, a pivotal study on the impact of anxiety on verbal WM processes has provided findings that implicate a central role for cognitive load in the interplay between anxiety and cognition (Vytal et al., 2012). Using *n*-back tasks of varying difficulty during periods of threat (shock) and safety (no shock), the authors found that performance was impaired by anxiety, but only when the task was easy or moderately challenging. When the task was difficult, anxiety was reduced, and performance did not differ between threat and safe conditions. As the first study to show that verbal WM is impaired by anxiety under low cognitive load, and that high-load verbal WM reduces anxiety, it highlights the importance of considering cognitive load in the study of emotion–cognition interactions. Together with key findings that suggest high-load spatial WM is susceptible to anxiety-related impairment (Shackman et al., 2006), these results indicate that although anxiety disrupts both verbal and spatial WM, the presence or degree of disruption is a function of both task modality and cognitive load. Studies that use a translational method of anxiety induction (threat of electric shock, used in conjunction with a no-shock safety condition) find robust anxiety-related performance deficits (Robinson et al., under review). Such studies have found that verbal (Vytal et al., 2012) and spatial WM (Lavric et al., 2003; Shackman et al., 2006) are impaired by anxiety, yet only low-load verbal WM is susceptible to disruption, whereas spatial WM is disrupted under high cognitive load. Thus, at 3-back, there is equitable performance under threat and safety when the task involves verbal stimuli, and impaired performance under threat, when the task involves spatial stimuli. However, it is unknown whether or not low-load spatial WM tasks are susceptible to disruption, and whether there is a differential impact of anxiety on verbal and spatial WM across a varying of cognitive load. In this study we sought to tease apart the impact of anxiety on both verbal and spatial WM, and determine whether or not task difficulty plays a role in this disruption. By determining the precise profile of WM impairment in anxiety, we will have a more comprehensive understanding of anxiety's impact on cognition. This knowledge can then be used to target the aberrant mechanisms that disrupt cognitive processes in pathological anxiety.

In the current experiment, threat of shock was used to induce sustained anxiety, and anticipatory anxiety was measured using acoustic startle reflex (eye blink) and subjective ratings. The startle reflex is an effective index of anxiety because it is robustly potentiated under anxious conditions, and this potentiation is thought to reflect priming of defense mechanisms in both humans and non-human animals (Davis, 1998; Grillon, 2002). On two separate sessions, participants performed a series of verbal and spatial *n*-back tasks of varying difficulty (1-back, 2-back, and 3-back) under threat and safe (no shock) conditions. Based on evidence that suggests low and medium-load verbal WM (Vytal et al., 2012) and other low-load tasks are disrupted by anxiety (Lavie, 2005) (but in opposition to the processing efficiency theory and the hemispheric asymmetry hypothesis), we predicted that both verbal and spatial low-load and medium-load WM (i.e., 1-back and 2-back) would be impaired under threat versus safe conditions. Here, we define impairment as a decrease in performance accuracy. However, we predicted that high-load spatial WM but not verbal WM would be affected by anticipatory anxiety (i.e., performance would be impaired during threat compared to safe conditions). These hypotheses are based on previous findings and predictions from both the two-component model and hemispheric asymmetry hypothesis that suggest high-load verbal and spatial WM are differentially impacted by anxiety. Finally, we predicted that individual differences in state anxiety (as indexed by anxiety potentiated startle and state anxiety ratings) would be negatively correlated with individual differences in performance, indicating that greater anxiety is associated with greater anxiety-related cognitive impairment. Along these same lines, we predicted that anxiety-potentiated startle would be positively correlated with anxiety (consistent with the claim that startle potentiation indexes anxiety). These predictions were all based on previous research suggesting that individual differences in anxiety predict impairment and startle potentiation is a robust index of anxiety (Shackman et al., 2006; Vytal et al., 2012). In summary, we expected that anxiety would differentially impact verbal versus spatial WM across increasing levels of cognitive load, such that (1) anxiety induction would impair lower-load (1-back and 2-back) but not higher-load (3-back) verbal WM, and (2) anxiety induction would impair both low and high-load spatial WM.

## Materials and methods

### Participants

Twenty-seven healthy individuals (13 males) received monetary compensation for their participation in the study. Participants were recruited for the study via online resources, paper flyers, and advertisements placed in local newspapers. Upon arrival, participants completed an intake evaluation consisting of a brief physical exam, urine screen, and a Structured Clinical Interview for DSM-IV (SCID; First et al., 1995). Exclusion was based on the following criteria: (1) past or current psychiatric disorder(s), (2) contraindicated medical condition, and (3) use of psychoactive medications or illicit drugs. Three participants were excluded because of equipment failure. The final group of participants consisted of 24 adults (11 males; mean age 29.5 years; age range: 18–46 years). Subjects provided written informed consent that was approved by the Combined Neuroscience Institutional Review Board of the National Institutes of Health.

### Stimuli and apparatus

All visual stimuli were presented on a PC using Presentation® software (Version 0.70, www.neurobs.com). Presentation® software was also used to control all electric shocks and startle probes via a commercial system (Contact Precision Instruments, London, United Kingdom). Shocks (up to 5 mA and 200 ms duration) were produced by a constant current stimulator and administered to the median nerve of the left wrist using two 6 mm Ag/AgCl electrodes. Shock level was determined independently for each participant using a shock workup procedure where the shock level began at 3.5 mA and was increased by increments of 0.2 mA until the subject rated the shock as highly uncomfortable, but still tolerable (*M* = 5.9; *SD* = 2) based on a 1–9 scale (1, not at all painful, to 9, extremely painful). Acoustic startle probes [40 ms, 103 dB(A), near instantaneous rise/fall times] were presented binaurally through over-the-ear headphones. The eye blink reflex was measured using two 6 mm Ag/AgCl electrodes (impedances below 15 kΩ) placed over the orbicularis oculi muscle under the left eye. Electromyographic (EMG) data were recorded by Psylab 7 software (Contact Precision Instruments, London, UK).

### Procedure

**Figure 1:**
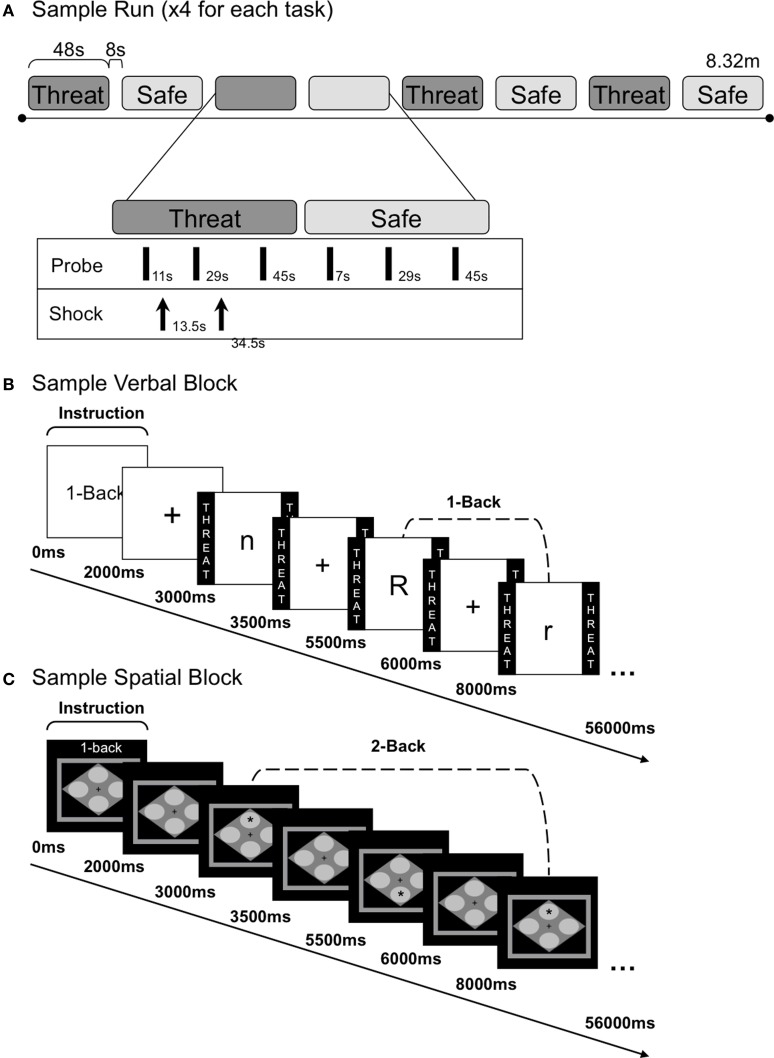
**Task run and block structure. (A)** Schematic diagram of a sample run with alternating threat and safe *n*-back blocks. During each *n*-back block, three acoustic probes were delivered. Shocks were delivered three times during each run (with 0–2 shocks each threat block). **(B)** Sample verbal 1-back block and internal trial structure. Each block began with an instruction screen, followed by a fixation cross. Eighteen letters were presented in succession during each block, separated by a 2 s ITI (fixation). Participants made a keyboard button press response for every letter presented; one button indicated a target letter (e.g., “r”) and another button indicated a distractor letter (e.g., “n” and “R”). During the view condition subjects attended to the letters without making a button press. **(C)** Sample spatial 2-back block and internal trial structure. Block structure was identical to the verbal *n*-back except that spatial stimuli were used. Participants made a keyboard button press response every time a star appeared in one of four locations; one button indicated a target location (e.g., “top”) and another button indicated a distractor location (e.g., “bottom”).

Procedures and task-design were identical to the those described in (Vytal et al., 2012), with the exception that in the current study, there were two sessions (counterbalanced order), one for the verbal *n*-back and the other for the spatial *n*-back (similar in design except that the location of a red star in one of four corners of a diamond was the target, as opposed to a letter). The basic layout was the same across sessions. To assess stable trait anxiety and experiment-induced state anxiety, all participants completed the Spielberger state-trait anxiety inventory (STAI; Spielberger et al., 1983) when they first arrived. Prior to the experiment, participants practiced all four levels (view, 1-back, 2-back, and 3-back) of each task (spatial and verbal) to reduce changes in performance as a result of learning. Participants indicated “same” or “different” with a keyboard button press based on the stimulus (verbal: letter, spatial: location) 1-back, 2-back, or 3-back from the current stimulus, or simply attended to the stimuli (“view” task) without making a response (see Figure 1B for a sample verbal block and Figure 1C for a sample spatial block). Following practice, participants were presented with nine startle probes every 17–20 s during a rest period in order to habituate initial startle reactivity.

Each session included four experimental runs, consisting of eight alternating threat and safe blocks (see Figure 1A for a sample run). Participants were reminded of the condition they were in [threat (at risk to receive shock) or safe (no shocks were delivered)] by colored borders (verbal: the word THREAT or SAFE was written inside, spatial: a red or blue border, represented threat and safe, respectively). Each run began with three habituation probes, followed by a 2-s instruction screen (e.g., “1-back”) and a 1000 ms fixation cross. Stimuli (18 in each block; 144 per *n*-back task; 288 threat, 288 safe) were presented for 500 ms each, separated by 2000 ms (±250 ms) fixation inter-trial intervals (ITIs). All verbal stimuli were presented in Arial, 48-point font in the center of the screen. Verbal *n*-back targets consisted of eight letters (B, F, K, H, M, Q, X, R), in both upper and lowercase to reduce reliance on perceptual similarity (as such, “b” and “B” were treated as identical targets). The spatial *n*-back target was a single asterisk, Arial 64-point font, successively presented in one of the four corners of a gray diamond (height: 7.5 cm, width: 10.8 cm), centered in the middle of the screen. During the ITI, twelve shocks (0–2 per threat block; 3 per run) and nine startle probes (every 17–20 s) were administered. To reduce sensitization effects of the shocks on startle, shocks preceded probes by at least 16 s, and followed probes with a mean latency of approximately 2 s. Shocks were only delivered during half of the threat blocks to prevent shock desensitization and to reduce potential effects of the shock itself (versus anticipation of the shock) on performance and startle. Blocks were separated by an 8 s inter-block interval.

### Data reduction and analysis

EMG data were sampled at 1000 Hz, filtered (30–500 Hz), rectified, and smoothed with a 20-ms time constant. Startle responses were defined as the peak magnitude of the eye blink reflex (20–100 ms after stimulus onset) relative to a 50-ms average baseline that immediately preceded the probe onset. Less than one percent of trials was excluded based on large baseline artifacts. T-score transformation was used to attenuate large inter-individual differences in raw reflex magnitude. Peak eye blink magnitudes were T-scored (across all conditions) and averaged within each condition for each subject. For correlation analyses, differential accuracy scores (threat–safe) and differential startle scores (threat–safe) were averaged across 1-back, 2-back, and 3-back blocks, resulting in an aggregate impairment score and aggregate startle potentiation score for each subject. To confirm that accuracy did not differ as a result of shock or probe administration, trials that preceded or followed shocks, and those that preceded or followed probes were analyzed separately. No differences were found and all trials were included in the final analysis. Trials where participants failed to respond before the next stimulus appeared on the screen (i.e., 2500 ms post-stimulus onset) were omitted. However, such omissions were uncommon and unsystematic. A series of binomial tests at the individual level confirmed that all participants included in the final analysis performed above chance. Alpha was set at 0.05 for all statistical tests. Repeated-measures ANOVAs, paired *t*-tests, and Pearson product moment correlation coefficients were all used to assess statistical significance. Greenhouse–Geisser corrections (GG-ε) were used for repeated-measures ANOVAs that involved factors with three or more levels.

### Psychometrics

We sought to examine the impact of anxiety on different modalities of WM (verbal and spatial) as well as different levels of cognitive load. As such, it was important to investigate psychometric equivalence so that discrete inferences about the differential effect of anxiety could be made in the absence of a double dissociation (where two or more experimental manipulations have opposing effects on two or more dependent variables) (Shackman et al., 2006). To determine psychometric equivalence we calculated discriminating power (Chapman and Chapman, 2001), which quantifies the sensitivity of a test to detect an experimental manipulation (or a group difference) between tasks where differences were found (see the results section for a full description of these findings). Discriminating power was computed by multiplying the accuracy variance across baseline (safe) runs by the reliability in accuracy (Cronbach's coefficient alpha) across those same runs. Comparison of verbal and spatial *n*-back discriminating power at high load (3-back) demonstrated that sensitivity did not differ between the two tests [*t*_(23)_ = 1.92, *p* > 0.05; *M* = 33.36 (verbal 3-back), *M* = 49.25 (spatial 3-back)]. This is critical because the differential impact of anxiety on cognitive load between verbal and spatial stimuli was present only in the high-load data. Further, we confirmed that task difficulty was equivalent between 3-back verbal and spatial WM tasks, [performance: *t*_(23)_ = −1.25, *p* = 0.226], suggesting that the tasks were similarly challenging and that impact of threat on 3-back spatial performance cannot be attributed to the fact that it was less challenging than verbal 3-back. In addition, comparison of verbal low-load to high-load discriminating power demonstrated that sensitivity was greater in the high-load task than in the low-load task [*t*_(23)_ = 5.39, *p* < 0.001; *M* = 33.36 (high-load), *M* = 12.72 (low-load)]. Given that low-load verbal WM tasks were found to be *less* sensitive than high-load verbal WM tasks, anxiety-related performance differences in low-load tasks cannot be attributed to greater discriminating power.

## Results

### Manipulation check

#### Anxiety

Without verification that our anxiety manipulation was successful, it would be difficult to clearly interpret any performance differences observed. Anxiety ratings in both studies indicated that subjects experienced more anxiety when they were at risk for shock [verbal: threat *M* = 5.5, safe *M* = 2.2, *t*_(23)_ = 7.6, *p* < 0.001; spatial: threat *M* = 5.3, safe *M* = 2.1, *t*_(23)_ = 10.1, *p* < 0.001]. In addition to self-report, we used startle magnitude to verify that threat of shock successfully induced anxiety. Startle was consistently potentiated by threat of shock, *F*_(1, 23)_ = 67.1, *p* < 0.0001, η^2^ = 0.75, confirming the manipulation. Moreover, anxiety-potentiated startle (threat–safe) was reduced by load [*F*_(3, 69)_ = 12.7, *p* < 0.001, η^2^ = 0.36], indicating that load decreased anxiety [confirmed by a linear trend: *F*_(1, 23)_ = 34.9, *p* < 0.0001, η^2^ = 0.60]. Startle did not differ as function of WM modality [Modality × Anxiety × Load = F_(3, 69)_ = 1.4, *p* = 0.252, η^2^ = 0.06].

#### Load

To verify that the *n*-back tasks of varying difficulty resulted in differing levels of cognitive load (reflected by performance), a repeated measures ANOVA was conducted across WM task modalities. The main effect of Load on performance was significant, *F*_(2, 46)_ = 113.0, *p* < 0.0001, η^2^ = 0.83, indicating that regardless of task modality and condition, overall WM performance differed across levels of cognitive load. A linear trend demonstrated that as load increased, performance decreased [*F*_(1, 23)_ = 200.3, *p* < 0.0001, η^2^ = 0.90], indicating that the more demanding tasks were in fact more challenging. To investigate this effect further, the results were considered separately for verbal and spatial tasks. Both verbal and spatial WM performance was impacted by Load, [*F*_(2, 46)_ = 50.8, *p* < 0.0001, η^2^ = 0.69 and *F*_(2, 46)_ = 50.1, *p* < 0.0001, η^2^ = 0.69, respectively], and planned comparisons indicate that as task difficulty increased, performance was progressively worse [verbal: 2-back performance was lower than 1-back, *t*_(23)_ = −3.9, *p* < 0.002; and 3-back performance was lower than 2-back, *t*_(23)_ = −5.8, *p* < 0.001, spatial: 2-back performance was lower than 1-back, *t*_(23)_ = −4.1, *p* < 0.001; and 3-back performance was lower than 2-back, *t*_(23)_ = −8.1, *p* < 0.001].

### Performance

**Figure 2:**
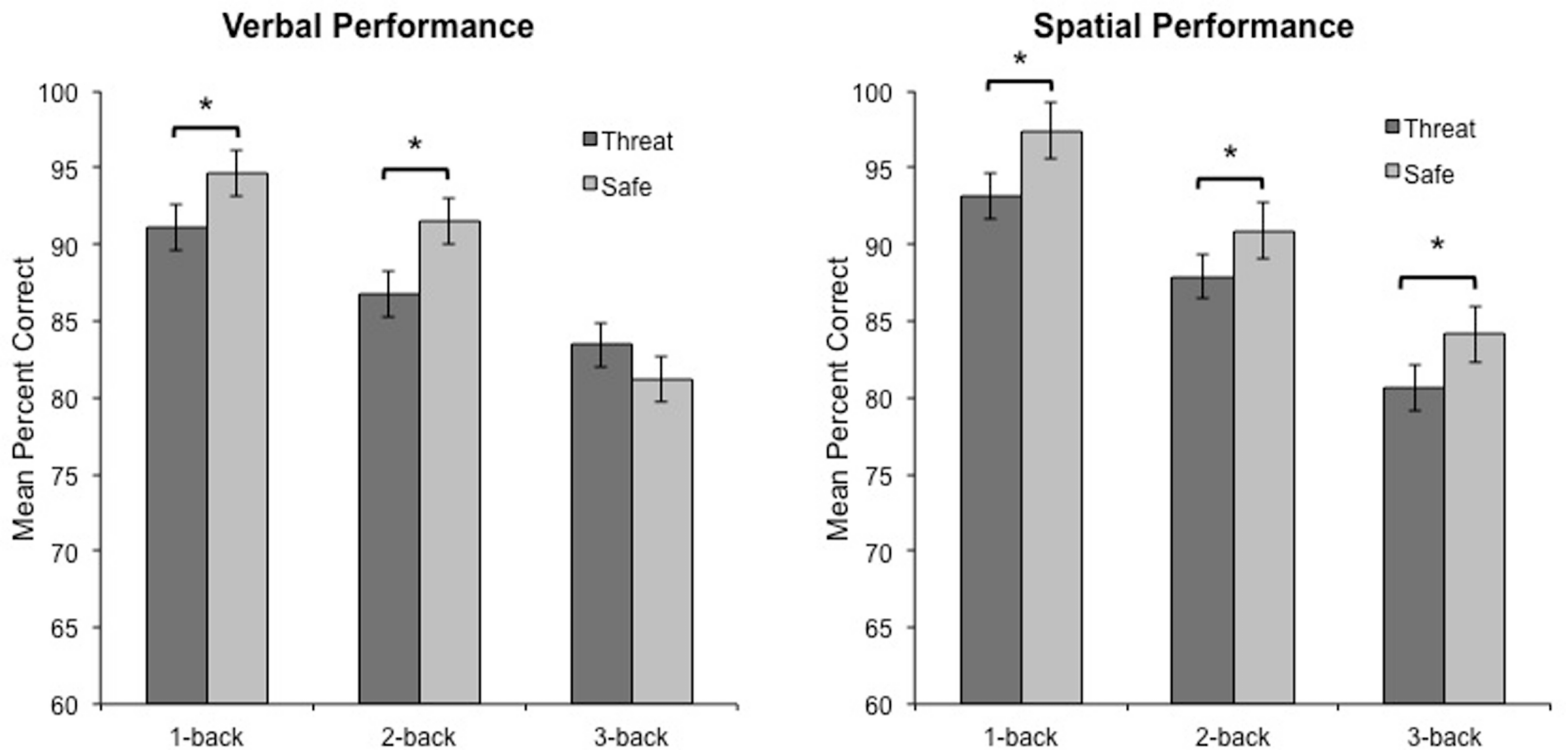
**Verbal and spatial *n*-back performance during threat and safe.** Verbal performance was impaired during threat compared to safe when participants were engaged in low-load tasks (1-back and 2-back), but not high-load tasks (3-back). In contrast, spatial performance was impaired during threat compared to safe when participants were engaged in any task, irrespective of difficulty. Error bars represent the within-subjects standard error for the repeated-measures general linear model (GLM) comparing different levels of Load under threat (dark gray bars) and safe (light gray bars) conditions separately. Within-subject standard error was calculated by dividing the square root of the mean standard error for the GLM divided by the square root of n (Masson, 2003). ^*^p < 0.01.

Consistent with our predictions, the critical three-way interaction between Modality, Anxiety, and Load, was significant, *F*_(2, 46)_ = 3.5, *p* < 0.04, η^2^ = 0.13, indicating that anxiety had a differential impact on overall WM performance across load. To decompose this interaction, performance data were analyzed separately for verbal and spatial WM tasks. For verbal WM, the interaction of Anxiety and Load was significant, *F*_(2, 46)_ = 6.9, *p* < 0.003, η^2^ = 0.23, reflecting the finding that 1-back and 2-back performance was impaired during threat as compared to safe [*t*_(23)_ = −2.5, *p* < 0.03, and *t*_(23)_ = −3.1, *p* < 0.006, respectively], but 3-back performance did not differ between conditions [*t*_(23)_ = 1.7, *p* = 0.101] (see Figure 2). Further, performance differences between threat and safe (i.e., threat–safe) were greater for 1-back and 2-back tasks as compared to 3-back [*t*_(23)_ = 2.2, *p* < 0.05, and *t*_(23)_ = 2.3, *p* < 0.04, respectively]. We confirmed that these findings were not driven by speed and accuracy tradeoffs, with RT analyses demonstrating that RT did not differ between threat and safe across Load, *F*_(2, 46)_ = 0.170, *p* = 0.845, and more specifically, RT differences (threat–safe) were not significantly different between low (1 and 2-back) and high load (3-back) [*t*_(23)_ = 0.2, *p* = 0.857, and *t*_(23)_ = 0.3, *p* = 0.741, respectively] (see Table 1 for RT means and standard errors of the mean). These findings suggest that in the case of verbal WM, lower-demand tasks are susceptible to disruption by induced-anxiety, whereas higher-demand tasks are not. In contrast to the verbal WM results, there was not a significant Anxiety × Load interaction for spatial WM, *F*_(2, 46)_ = 0.31, *p* < 0.738, η^2^ = 0.01. However, there was a significant main effect of Anxiety on performance, *F*_(1, 23)_ = 18.8, *p* < 0.001, η^2^ = 0.449, indicating that spatial WM performance was impaired overall during threat as compared to safe, regardless of task difficulty (see Figure 2). This finding indicates that under both low and high cognitive load, an anxiogenic context impaired spatial WM. As with verbal WM, we confirmed that RT did not differ between threat and safe across Load for spatial WM, *F*_(2, 46)_ = 2.6, *p* = 0.085.

**Table 1:** **Mean reaction time for verbal and spatial working memory as a function of experimental condition and cognitive load**.

	**1-back**	**2-back**	**3-back**
**VERBAL**			
Threat	703 (32)	787 (41)	793 (44)
Safe	712 (35)	778 (40)	792 (40)
**SPATIAL**			
Threat	721 (31)	834 (48)	846 (42)
Safe	756 (42)	816 (47)	870 (48)

Note: Standard errors of the mean appear in parentheses to the right of each mean.

### Correlations

**Figure 3:**
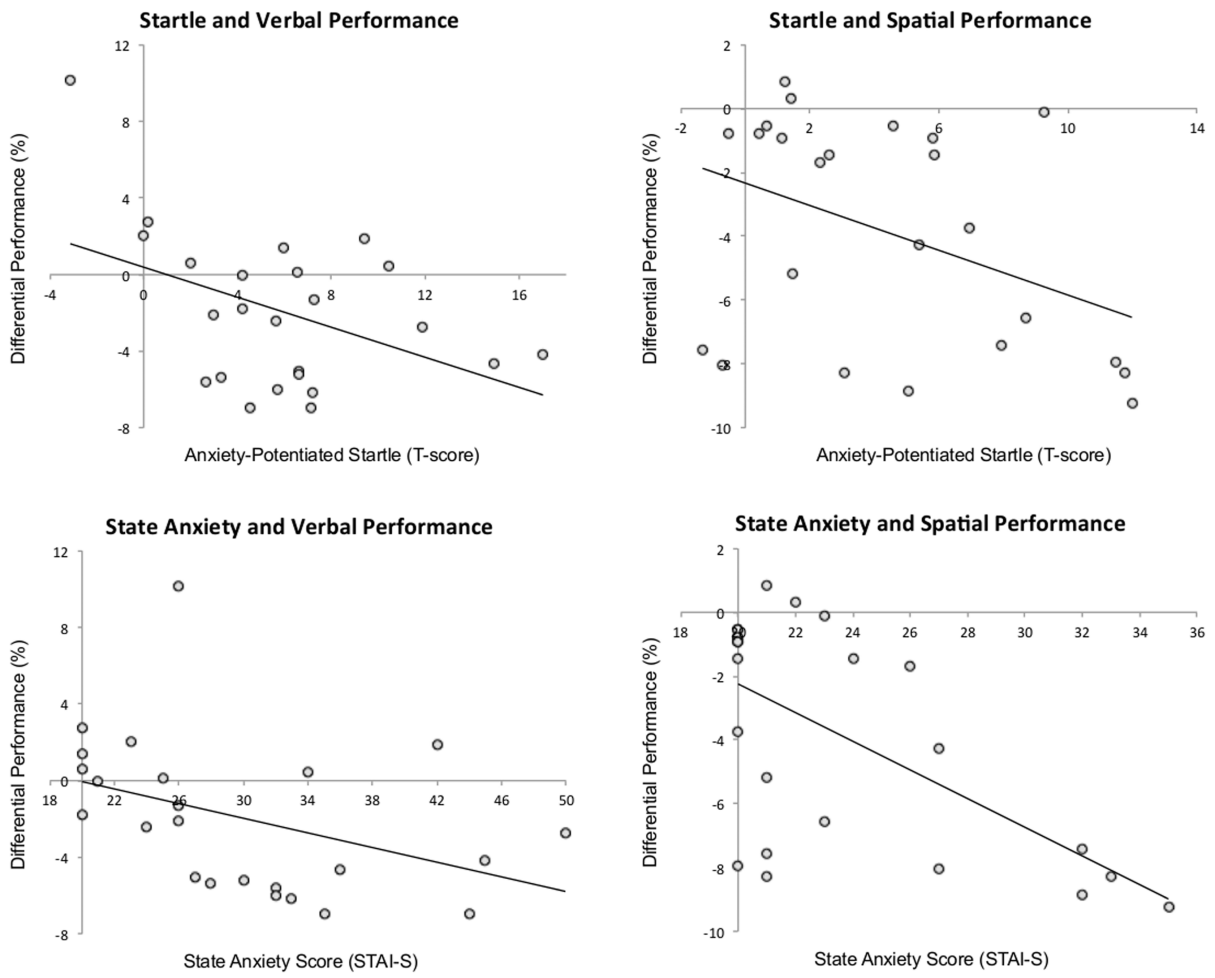
**The relationship among anxiety-potentiated startle, performance (threat–safe), and state anxiety.** Startle potentiation was negatively correlated with verbal and spatial working memory performance, and state anxiety was negatively correlated with verbal and spatial working memory performance. *p* < 0.05 for all correlations.

There was a negative correlation between anxiety-potentiated startle and differential performance under threat (threat–safe) for both verbal, *r* = −0.44, *p* < 0.04, and spatial WM, *r* = −0.41, *p* < 0.05, demonstrating that increased startle potentiation was associated with increased WM impairment (see Figure 3 for scatterplots). In line with this, there was also a negative correlation between state anxiety and differential performance under threat in verbal, *r* = −0.41, *p* < 0.05, and spatial WM, *r* = −0.61, *p* < 0.01, reinforcing the idea that high levels of anxiety were associated with greater verbal WM impairment. Additionally, we confirmed that anxiety-potentiated startle was a good index of anxiety (as assessed by state anxiety scores) in both tasks (verbal: *r* = 0.66, *p* < 0.01; spatial: *r* = 0.47, *p* < 0.03). Together, these findings suggest that anxiety is a strong predictor of threat-related verbal WM impairment.

To further unpack the interaction of Modality and Load on anxiety-related WM impairments, and to address the prediction that verbal WM will be more sensitive to parametric modulation of task difficulty, we subtracted the difference between threat and safe during high load (performance and startle), from the difference between threat and safe during low load (performance and startle) and conducted a correlation analysis. As predicted, we found that the differential anxiety-potentiated startle scores were negatively correlated with the differential performance scores (1-back minus 3-back) for verbal WM (*r* = −0.58, *p* < 0.02), but not spatial WM (*r* = −0.17, *p* = 0.44). Moreover, Fisher's *z*-test confirmed that these two correlations were significantly different, *z* = 1.7, *p* > 0.05. This negative correlation indicates that the more anxiety-potentiated startle was reduced from 1-back to 3-back (i.e., indicating reduction in anxiety), the better the performance improvement was from 1-back to 3-back in threat versus safe. This suggests that load is an important manipulation in characterizing the impact of anxiety on verbal WM performance, and that it is less critical in characterizing the impact of anxiety on spatial WM performance.

## Discussion

### General discussion and overview of findings

Anxiety helps maintain a state of readiness. It facilitates threat processing and defensive responding but it also prompts cognitive changes. Studying these changes using dual-task paradigms may help to clarify behavioral performance under stress (test anxiety, decision making/planning in the battlefield or during an emergency) and emotion regulation mechanisms. In addition, this line of research can identify specific cognitive deficits associated with anxiety and anxiety disorders. Concerning the latter, the present study suggests that the cognitive and defensive components of anxiety interfere with WM tasks but to a different degree, such that anxious apprehension has more of a domain-general impact on WM, high-load verbal WM engages top-down control mechanisms that abolish anxiety-related disruption, and spatial WM is more vulnerable to the effects of anxious arousal.

As predicted, induced-anxiety impaired both verbal and spatial WM processes, but anxiety had a different impact on performance when cognitive load was considered. Results demonstrate that low-load verbal WM is more susceptible to anxiety-related disruption and spatial WM is disrupted regardless of task difficulty. Well-validated measures of anxiety (state anxiety and startle potentiation) strongly predicted variability in performance impairment, underscoring the specificity of these effects. These novel results provide a framework for understanding the interaction between anxiety and two distinct modalities of WM, by emphasizing the effect of cognitive load on performance. Further, these findings are in line with the two-component model (Vytal et al., 2012), which proposes a differential effect of anxiety on verbal versus spatial WM based on competition over two separable neural circuits [a conceptual distinction that was proposed but not substantiated in prior work (Shackman et al., 2006)].

### Integration with current theories

While there is clear support for the two-component model of anxiety, the processing efficiency theory (Eysenck and Calvo, 1992) receives only limited support and the hemispheric asymmetry hypothesis (Shackman et al., 2006) serves as only a partial explanation for these findings. First, our data do not support a key prediction of processing efficiency theory [and its offshoot, attentional control theory (ACT) (Eysenck et al., 2007)], namely, that anxious worry increases RT. However, although we did not find RT differences between any of our conditions, we found performance differences in partial support of these theories. Processing efficiency theory and ACT predict that anxiety impairs high-load WM is impaired when a subject is anxious, regardless of the task modality, yet our findings suggest that only spatial WM is disrupted under high cognitive load. Similarly, the hemispheric asymmetry hypothesis proposes that processes which rely heavily on the right hemisphere [e.g., spatial attention (Corballis et al., 2002; Manoach et al., 2004)] are disrupted by anxiety because anxious arousal consumes right hemisphere resources (Clark et al., 2003; Dalton et al., 2005). This prediction holds true to an extent; spatial WM is disrupted by anxiety (Lavric et al., 2003; Shackman et al., 2006), and it is plausible that this disruption is the result of competition for shared resources between spatial attention and automatic priming of defensive mechanisms. However, there is now ample evidence to suggest that anxiety also impairs verbal WM (Markham and Darke, 1991; Ikeda et al., 1996; Vytal et al., 2012), leaving that mechanism of impairment undefined.

The two-component theory of anxiety fills this explanatory gap by describing a specific mechanism for verbal WM disruption. Anxiety, which is comprised of a cascade of physiological and neural responses, is best characterized by two separable components: (1) an anxious apprehension component (Heller et al., 1997) that engages executive resources and includes anxiety-related cognitive processes like worry, and (2) an automatic preparatory response that primes defensive mechanisms (Lang et al., 1998), increases perceptual sensitivity (Cornwell et al., 2011), and enhances autonomic arousal (e.g., increases in heart rate and blood pressure) (Bandura, 1988). Although this distinction is not new (Heller et al., 1997) the application of such framework to modality-specific WM disruption is novel. The anxious apprehension component and automatic preparatory component engage separable neural circuits (Nitschke et al., 1999), and as a consequence, they have a differentiable impact on processes that share these same respective neural resources. Established neural correlates of verbal and spatial WM (D'Esposito et al., 1998) overlap with the anxious apprehension and preparatory component circuitry respectively. With respect to verbal WM (D'Esposito et al., 1998; Kalisch et al., 2006) and anxious apprehension (Engels et al., 2007; Paulesu et al., 2010), such regions include bilateral dorsal, medial, and left ventral PFC, and with respect to spatial WM (Manoach et al., 2004) and the preparatory component (Clark et al., 2003; Dalton et al., 2005), such regions include right dorsal/mid and ventral PFC.

Thus, it appears that when anxiety promotes adaptive responses to threat [e.g., increased heart rate (Bandura, 1988), potentiation of visual (Shackman et al., 2011) and auditory perception (Cornwell et al., 2007), amplified attention to emotionally negative stimuli (Robinson et al., 2012)], such changes commandeer neural resources that are critical to WM maintenance. These resources can be reappropriated by increasing the demands of a verbal task, in turn reducing anxiety and normalizing performance in the face of threat (Rapee, 1993; Vytal et al., 2012). Of note, both easy (e.g., 1-back) and moderately difficult (2-back) verbal WM tasks are disrupted by anxiety, indicating that even when there is partial competition for resources, anxiety continues to control shared neural real estate. Only when task demands increase sufficiently to significantly (or completely) consume resources, is the effect of anxiety on performance abolished. Top-down emotional control mechanisms and domain-general WM are mediated by the same neurocognitive mechanisms [e.g., lateral PFC (Brodmann area 9) and dorsomedial PFC (Brodmann area 6); for meta-analytic reviews of WM neuroimaging studies see Owen et al., 2005; Nee et al., 2013, for explicit emotional regulation studies see Ochsner et al., 2004; Kim and Hamann, 2007; Diekhof et al., 2011], suggesting that down-regulation of anxiety may occur through either conscious or incidental regulation. However, increasing the demand of a spatial task does not result in normalized performance; accuracy is still impaired even under high-demand spatial WM maintenance. We propose that there are three potential reasons for this sustained impairment. First, in line with the hemispheric asymmetry hypothesis, anxiety shares a greater amount of critical resources with spatial WM processes [including spatial attention (Cornwell et al., 2008), perception, and maintenance] and therefore has a greater impact on spatial WM. Second, physiological changes associated with defensive readying (i.e., changes in spatial attention, visual acuity etc.) are more protracted (Bonanno et al., 1995) and may be less frequently and more circuitously subject to explicit regulation than cognitive responses to stress (top-down control of lower-order subcortical processes that promote survival may be more difficult than cortical control of other higher-order cortical responses). As a consequence, difficult spatial WM processes that share critical mechanisms with defensive preparations may continue to be disrupted. Third, although cognitive load can reduce anxiety and threat-related distraction (Vytal et al., 2012), defensive mechanisms remain intact under high load to promote survival, and as a consequence spatial WM impairment associated with these mechanisms may also persist.

### Implications for pathology

Clinical anxiety is associated with known disruptions in the cognitive domain, including WM (Lucas et al., 1991; Boldrini et al., 2005) spatial perception (Jacob et al., 1985; Simon et al., 1998), and spatial navigation (Cohen et al., 1996; Mueller et al., 2009) among others. These disruptions, however, are accompanied by facilitation in related domains, like visual threat detection (Bar-Haim et al., 2007), which may be supported by modulation of early sensory processes in anxiety disorders (Morgan III and Grillon, 1999; Ge et al., 2011). It follows that the greatest negative impact of this facilitation is on tasks that share resources with processes that support threat detection (e.g., a spatial WM task that requires rapid detection and sustained maintenance of perceptual information). Our findings support this claim, by demonstrating that anxiety-induction in healthy individuals results in robust impairment of spatial WM. These parallels also validate the use of threat of shock to model pathological anxiety in healthy individuals (for a review on the similarities between findings from threat of shock paradigms and pathological anxiety, see Robinson et al., under review). It is important to note however, that in addition to changes in spatial attention and perception, pathological anxiety [in particular, generalized anxiety disorder (GAD) (Brown et al., 1992)] is also associated with higher-order cognitive processes like excessive worry that involve verbally-based changes in thought (Borkovec and Inz, 1990). Here, our findings add additional insight into WM disruption; easy verbal WM task performance is impaired by anxiety, but more difficult verbal tasks result in normalized performance. These findings have critical implications for understanding the nature of disruption (as described earlier), detecting anxiety-related impairment(s), and improving treatment of different anxiety disorders.

Although anxiety can be viewed as a continuous psychological construct, with a threshold of severity separating health and pathology, anxiety disorders are comprised of categorically-separable manifestations of anxiety, with markedly different symptom profiles^1^. GAD, for example, is characterized by excessive worry (Borkovec and Inz, 1990; Brown et al., 1992), whereas panic disorder (PD) is characterized in terms of somatic symptoms that center on cardiovascular changes (Katon, 1984). By focusing on central symptoms of each patient and identifying the etiology of such symptoms, appropriate treatment methods can be better applied. For example, overloading the verbal WM system is shown to reduce threat-related cognitive distraction and reduce anxiety-related WM impairments (Vytal et al., 2012). Techniques like cognitive behavioral therapy can take advantage of this and integrate similar procedures in the treatment of patients with GAD. On the other hand, individuals with somatic anxiety symptoms (e.g., PD) may exhibit greater spatial impairments including orientation (Jacob et al., 1985; Simon et al., 1998) and WM (Boldrini et al., 2005), thus identifying cognitive markers for the disorder. In contrast to overloading the WM system, effective treatment for PD may include addressing the somatic aspects of the disorders with pharmacological interventions that alter noradrenergic function [e.g., imipramine or alprazolam (Charney et al., 1986)], compounds that selectively gate communication between amygdala and brainstem known to support physiological responses to threat [the medial part of the central nucleus of the amygdala and the dorsal vagal complex (Viviani et al., 2011)], and therapeutic interventions like progressive relaxation that target somatic symptoms (Davidson, 1978).

### Strengths and limitations

A major strength of the study was the use of a within-subject design, which increases statistical efficiency (i.e., the ability to detect an effect), and decreases the potential that group differences are driven by the individuals that comprise it rather than the experimental manipulation (because the groups are made of identical participants). Another advantage of this design was the use of an anxiety-induction manipulation where (1) subjects could serve as their own controls and (2) the emotional *state* of anxiety could be isolated without the complications of pathology or trait variable that may or may not index the state of interest. Further, the parametric nature of the design afforded the detection of different impairment patterns in spatial versus verbal WM across levels of cognitive load, an effect that is novel and one that holds important theoretical implications.

Limitations of the study included the type of stimuli used, the lack of a direct measure of anxious apprehension, and the use of a healthy sample. The stimuli used in the verbal and spatial tasks were not identical, as those in some previous studies were (Lavric et al., 2003; Shackman et al., 2006), which could account for a portion of the variability in performance between the two tasks and could introduce uncertainty in the strategies used (verbal or spatial) in each task. However, (1) the tasks were psychometrically matched, suggesting they were similarly susceptible to anxiety-related disruption, (2) subjects reported using verbal strategies (e.g., subvocalization) in the verbal task and spatial strategies (e.g., mentally superimposing visual representations), strategies that were only successful for the task in which they were used, suggesting that the tasks successfully tapped verbal and spatial WM, and (3) by using different stimuli, participants were not required to switch strategies on the same set of stimuli, possibly introducing interference effects and changing the nature of the task. Other task-specific potential limitations include the issue of equating difficulty between the verbal and spatial tasks in order to accurately interpret the differential effect on performance. To address this, we examined baseline (i.e., during safe) performance and found no difference between verbal and spatial WM tasks. These findings suggest that task difficulty did not differ between modalities because cognitive effort and performance accuracy was equivalent. In addition, we make claims about the presence of anxious apprehension without presenting a direct measure of this component. While the Penn State Worry Questionnaire (Molina and Borkovec, 1994) may be a viable measure, future studies should also obtain online worry ratings for comparison between experimental conditions. Finally, it is important to note that our sample consisted of healthy individuals, not anxiety patients, and any conclusions drawn regarding pathological anxiety or clinical interventions should be interpreted with caution. Although we identified mechanisms of impairment, these mechanisms may be manifested differently in anxious individuals. Future research should include patient samples to identify and contrast pathological anxiety-related cognitive impairment.

### Conclusions and future directions

Previous research has struggled to identify the mechanisms of cognitive impairment in anxiety, despite the obvious presence of cognitive disruption in both state and clinical anxiety. Most individuals have experienced intense apprehension, along with sweaty palms and heart pounding, that can serve to debilitate them during goal-directed behavior such as giving a public speech. Patients who suffer from social anxiety are crippled when entering a jovial room full of party-guests, or an important staff meeting at work. The impact of anxiety on cognition is undeniable. Here we systematically pinpoint where anxiety disrupts verbal and spatial WM processes, highlighting the importance of task modality and cognitive load. In sum, our findings demonstrate that (1) anxiety disrupts both verbal and spatial WM, (2) that this disruption is only present in low and medium-load verbal WM, and (3) that this disruption is present in spatial WM regardless of task difficulty. We propose that there are separable neural mechanisms of disruption that arise from competition with two different components of anxiety (anxious apprehension and priming of defensive mechanisms), resulting in the aforementioned pattern of impairment. Future research should investigate the neural underpinnings of this disruption to verify these mechanisms of impairment and extend the investigation to patient populations so that individual differences in anxiety-related impairment can be evaluated as a potential risk factor in the development of pathology.

### Conflict of interest statement

The authors declare that, except for income received from the primary employer, no financial support or compensation has been received from any individual or corporate entity over the past 3 years for research or professional service and there are no personal financial holdings that could be perceived as constituting a potential conflict of interest.

## Footnotes

^1^However, it is important to note that comorbidity across different internalizing disorders suggests that a common construct links them (Watson, 2009).
